# New marine data and age accuracy of the Romualdo Formation, Araripe Basin, Brazil

**DOI:** 10.1038/s41598-020-72789-8

**Published:** 2020-09-25

**Authors:** Robbyson Mendes Melo, Juliana Guzmán, Débora Almeida-Lima, Enelise Katia Piovesan, Virgínio Henrique de Miranda Lopes Neumann, Ariany de Jesus e Sousa

**Affiliations:** 1 Laboratory of Applied Micropaleontology (LAGESE/LITPEG), Department of Geology, Federal University of Pernambuco, Recife, PE Brazil; 2 PETROBRAS/CENPES/PDGEO/BPA, Ilha do Fundão, Rio de Janeiro, RJ Brazil

**Keywords:** Stratigraphy, Geology, Palaeontology, Sedimentology

## Abstract

A combined biostratigraphic and palaeoecological study of foraminifera, ostracodes and microfacies was carried out on the Aptian in the Sítio Sobradinho section of the Araripe Basin, northeast Brazil. The analysed section represents a deepening-upward sequence with mid-ramp shoal and outer ramp to basin facies associations on a mixed siliciclastic-carbonate marine ramp. The analysed rocks are dominated by Early Cretaceous planktic foraminifera (*Hedbergella aptiana, H. praelippa, H. sigali*, *Blesfucuiana* cf. *cumulus, Microhedbergella miniglobularis, Gorbachikella* cf. *kugleri, Pseudoguembelitria blakenosensis, Globigerinelloides clavatus*, *Globigerinelloides* aff. *aptiensis*, *Gubkinella* sp. and *Loeblichella* sp.). Ostracoda fauna is composed mainly of *Pattersoncypris crepata* and *Pattersoncypris micropapillosa*. The occurrence of *P*. *crepata* associated with the Aptian planktic foraminifera demonstrates the potential of this ostracode species to date this interval. The planktic foraminifera from the upper Aptian (*Microhedbergella miniglobularis* Zone) of the Araripe Basin show characteristical Tethyan affinities.

## Introduction

**Figure 1:**
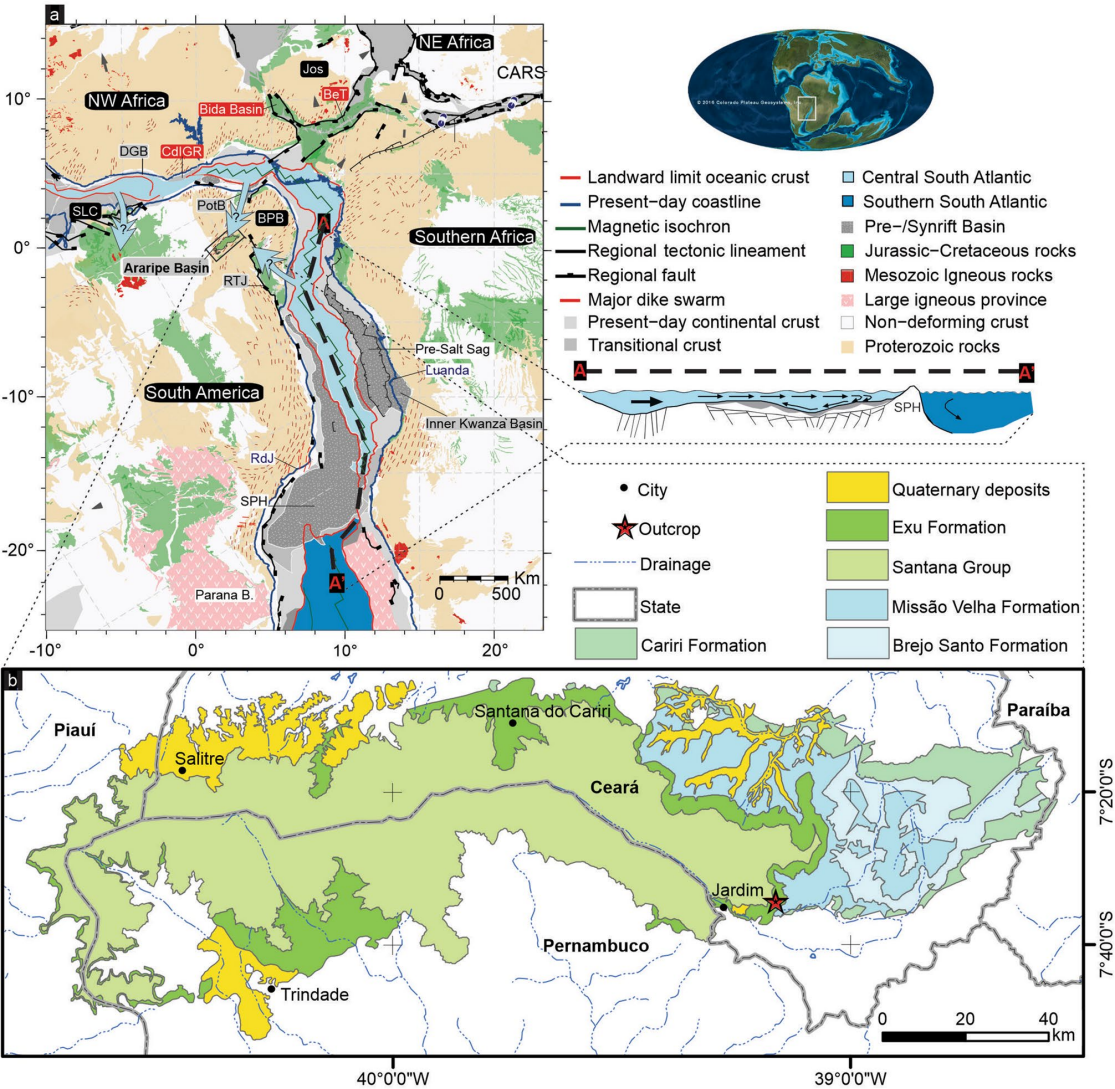
Location of the study area. (**A**) Palaeogeographical map of South America and Africa during the break-up of Gondwana by the late Aptian (~ 113 Ma) modified after the 115 Ma and 110 Ma plate tectonic reconstructions of Heine et al.^28^ with Africa fixed at present-day coordinates. Rigid lithospheric blocks are denoted by black labels: South African plate, *BPB* NE Brazilian Borborema Province plate, *Jos* Jos Plateau sub-plate, *SLC* Sao Luis Craton block. Actively extending basins are indicated by labels with a red background; post-rift basins are indicated by labels with a light grey background; the Araripe Basin is highlighted in the black box; abbreviations: *CARS* Central Africa Rift System, *BeT* Benue Trough, *CdIGR* Cote d’Ivoire/Ghana Ridge and associated marginal basins; *DGB* Deep Ghanian Basin, *PotB* Potiguar Basin, *RTJ* Recôncavo, Tucano and Jatobá Basins. Other abbreviations: *RdJ* Rio de Janeiro, *B*. basin, *SPH* São Paulo High. The A–A’ profile illustrates a longitudinal section extending from the equatorial region in the Central South Atlantic to the Southern South Atlantic, between which is the barrier of the São Paulo High comprising the Rio Grande Rise and the Walvis Ridge, after Carvalho et al.^115^. The global palaeogeographical sample map was obtained from Colorado Plateau Geosystems https://deeptimemaps.com/. (**B**) Geological map of the Araripe Basin created using ESRI ArcMap v.10.3 software (https://www.esri.com/en-us/arcgis/products/arcgis-pro/overview) with metadata acquired from the online Geosciences Service of the Geological Service of Brazil-CPRM. The complete figure was composed by Juliana Guzmán using CorelDRAW X7 software (https://www.coreldraw.com).

Several intracratonic and marginal basins of eastern South America and western Africa record the opening of the South Atlantic as a large Jurassic–Cretaceous intraplate rift zone during the break-up of western Gondwana. In the Borborema Province (BP) of northeast Brazil, the Araripe Basin aulacogen, the most extensive of the basins located south of the E–W Patos Shear Zone, is elongated in the E–W direction (Fig. 1), but its general structure is made of NE–SW asymmetric grabens. The Araripe Basin is known worldwide by its late Early Cretaceous fossil Konservat-Lagerstätten of the Crato and Romualdo formations, Santana Group^1,2^. Plants, including angiosperms, insects, fishes, terrestrial and flying reptiles, and abundant microfossils such as ostracodes and palynomorphs have been recovered from the continental strata of these units^3–11^. Marine fossils exemplified by dinoflagellates, foraminifera, fishes, echinoids and mollusks are also found in the Romualdo Formation^11–15^ and record the establishment of a marine ingression, on the aborted intraplate Araripe rift.

The sedimentological record of Mesozoic rifting in the Araripe Basin overlies the syneclise deposits^16^ of the Palaeozoic Cariri Formation^17^ and the Precambrian rocks of the Transversal Zone of the BP^18^. The Mesozoic stratigraphic sequences have been grouped according to the different rift tectonic stages; here, we considered three main stages. The Upper Jurassic Brejo Santo and Missão Velha formations are linked to the initial rifting stages in a large sedimentary basin called the Afro-Brazilian Depression^19–21^. The rifting climax recording the development of half-grabens comprises the fluvio-deltaic-lacustrine strata of the Berrisian Abaiara Formation^21^. The post-rift stage is divided into two sequences: the lower sequence corresponds to the lower Cretaceous Santana Group ranging from the base to the top of the Barbalha, Crato, Ipubi and Romualdo formations, and the upper sequence corresponds to the alluvial units of the Araripe Group, namely, the Araripina and Exu formations^22^. As with all intracratonic and marginal basins in Brazil, the rift-related sedimentary succession in the Araripe Basin displays a continuous record of ostracodes and palynomorphs throughout the Mesozoic, enabling the biostratigraphy and palaeoenvironmental evolution of this basin to be interpreted^6^. Fluvial and lacustrine environments characterize most of the strata that comprise the Araripe Basin Mesozoic record, but at the top of the succession, the evaporitic Ipubi Formation and the marine fossil content of the Romualdo Formation allow the recognition of the Aptian marine transgression into the basin and the posterior retreat registered by the Araripe Group. Subsurface data of the Romualdo Formation are quite scarce, and hence, vertical stratigraphic sections in outcrops are essential to better understand its stratigraphy. The Romualdo Formation comprises a wide range of lithologies, including stratified conglomerates, fine- to medium-grained sandstones, laminated limestones, marls, shales and coquinas, which characterize a coastal to marine environment^22^. Along the slopes of the Araripe Plateau are known good outcrops of this unit, such as the Sítio Sobradinho outcrop, which reaches a thickness of ~ 100 m and is the most representative outcrop showing the vertical stratigraphic section of the Romualdo Formation, allowing detailed studies. According to Custódio et al.^23^, the absence of the Ibupi Formation in the Sítio Sobradinho section causes nearshore deposits to rest unconformably atop the carbonate-siliciclastic facies of the Crato Formation. Four facies associations are related to a transgressive system track stacking pattern and the posterior highstand system track^23^.

Many discrepancies remain among the studies regarding how seawater reached the Araripe Basin^24–27^, as illustrated in the Fig. 1. Tectonic and geodynamical models show the opening of the South Atlantic Ocean beginning from the south. According to Heine et al.^28^, SW-directed extension had already started during the latest Jurassic in the southern part of the South Atlantic Rift System; in addition, break-up and seafloor spreading started near 138 Ma (mid-Berriasian), during which time E–W-directed extension between South America and Africa occurred at very low extensional velocities until the Hauterivian (~ 126 Ma), when rift activity in the equatorial Atlantic started to increase significantly with significant rotation towards NE–SW. From the base of the Aptian onwards, diachronous lithospheric breakup occurred along the central South Atlantic Rift, and the final break-up between South America and Africa occurred in the Santos-Benguela segment at approximately 113 Ma and in the equatorial Atlantic between the Ghanaian Ridge and the Piauí-Ceará margin at 103 Ma^28^. On the other hand, eustasy studies demonstrate that the sea level remained higher than the present-day mean throughout the Cretaceous, though the Aptian and early Albian eustatic sea level went into a long period of stasis, during which the sea level varied only by a relatively moderate amplitude (between 25 and 75 m)^29^.

In palaeoclimatic terms, the Aptian (∼ 125 to ∼ 113 Ma) was characterized by climatic changes and profound environmental perturbations, including Oceanic Anoxic Event (OAE) 1a (∼ 120 Ma), representing a global phenomenon of organic matter burial in oxygen-depleted oceans^30–34^. Apart from the terminal Cretaceous extinction, the planktic foraminiferal turnover across the Aptian/Albian boundary interval is the most dramatic event in the Cretaceous evolutionary history of planktic foraminifera, with a change from large-sized and heavily ornamented species in the latest Aptian to small-sized, globigeriniform specimens in the earliest Albian^35–38^. In the Araripe Basin, the occurrence of foraminifera associations has previously been recognized, inferring a marine ingression into the Araripe Basin recorded in the Romualdo Formation, but their taxonomy and distribution are not documented in detail^39,40^. On the other hand, palynoforaminifers (chitinous linings present in palynological preparations) are usually rare and related to benthic taxa^41–44^. Goldberg et al.^44^ especially recovered palynoforaminifera in the infra-Ipubi strata of the Araripe Basin, indicating that marine ingression occurred before evaporitic deposition. The ostracodes from the Araripe Basin have received the attention of several authors due to their abundance, diversity and excellent preservation^6,45,46^, including an unusual record of ostracodes with phosphatized appendages and eggs in deposits from the Romualdo Formation^3,47–49^. The absence of reliable index fossils has led to constant debates about the age of the Romualdo Formation, being positioned on the Alagoas local stage (Ostracoda Zone RT-011) in the Aptian–Albian^17,22^. To constrain the chronostratigraphic position of this interval, we report robust marine data from the Romualdo Formation represented by a set of microfossils, including planktic and benthic foraminifera, ostracodes and calcispheres of Early Cretaceous age from the Araripe Basin. The significance of this study lies in the calibration of Aptian planktic foraminiferal events with ostracode species.

## Methods

A lithostratigraphic section of the maximum flooding zone of the Romualdo Formation at the Sítio Sobradinho outcrop was performed. According to the lithological variation, thirteen samples were collected for micropalaeontological analysis, and six petrographic slides were prepared for microfacies analyses. Petrographic analysis was carried out with a Zeiss Axio Scope.A1 microscope equipped with a Zeiss AxioCam MRc camera at the Applied Micropaleontology Laboratory (LMA) of the Federal University of Pernambuco, Brazil. Microphotographs of petrographic slides and selected carbonate microfossils were obtained from a Phenom XL scanning electron microscope (SEM) at the LMA.

For the studies of carbonate microfossils, approximately 60 g of sediment was used, and mechanical disaggregation of the lithic samples was performed on smaller fragments (⁓5 mm), followed by immersion in water. After a period of 24 h, the sample was washed in water using different sizes of sieves (> 500 μm, > 250 μm, > 180 μm, > 63 μm and > 45 μm) and dried at 50 °C. This technique allows the extraction of planktic foraminifera, as well as benthic foraminifera, ostracodes, and other microfossils, without destroying or corroding the microfossils. Specimens that remained with the aggregated sediment were treated in an ultrasound bath at variable times. Picking was performed under a Zeiss Stemi 305 stereomicroscope at all fractions, from > 250 μm and > 180 μm fractions, all specimens were collected. The smallest fractions samples (> 63 μm and > 45 μm) displayed high abundance and were quartered; the picking process reached 300 specimens from each fraction sample. The age of the sedimentary section studied here was attributed based on the known biostratigraphic ranges of the recovered species using the scheme by Huber and Leckie^50^ and Petrizzo et al.^51^ as the basis. The specimens presented here were deposited in the LMA, under the collection numbers LMA-00029 to LMA-00073.

## Results

**Figure 2:**
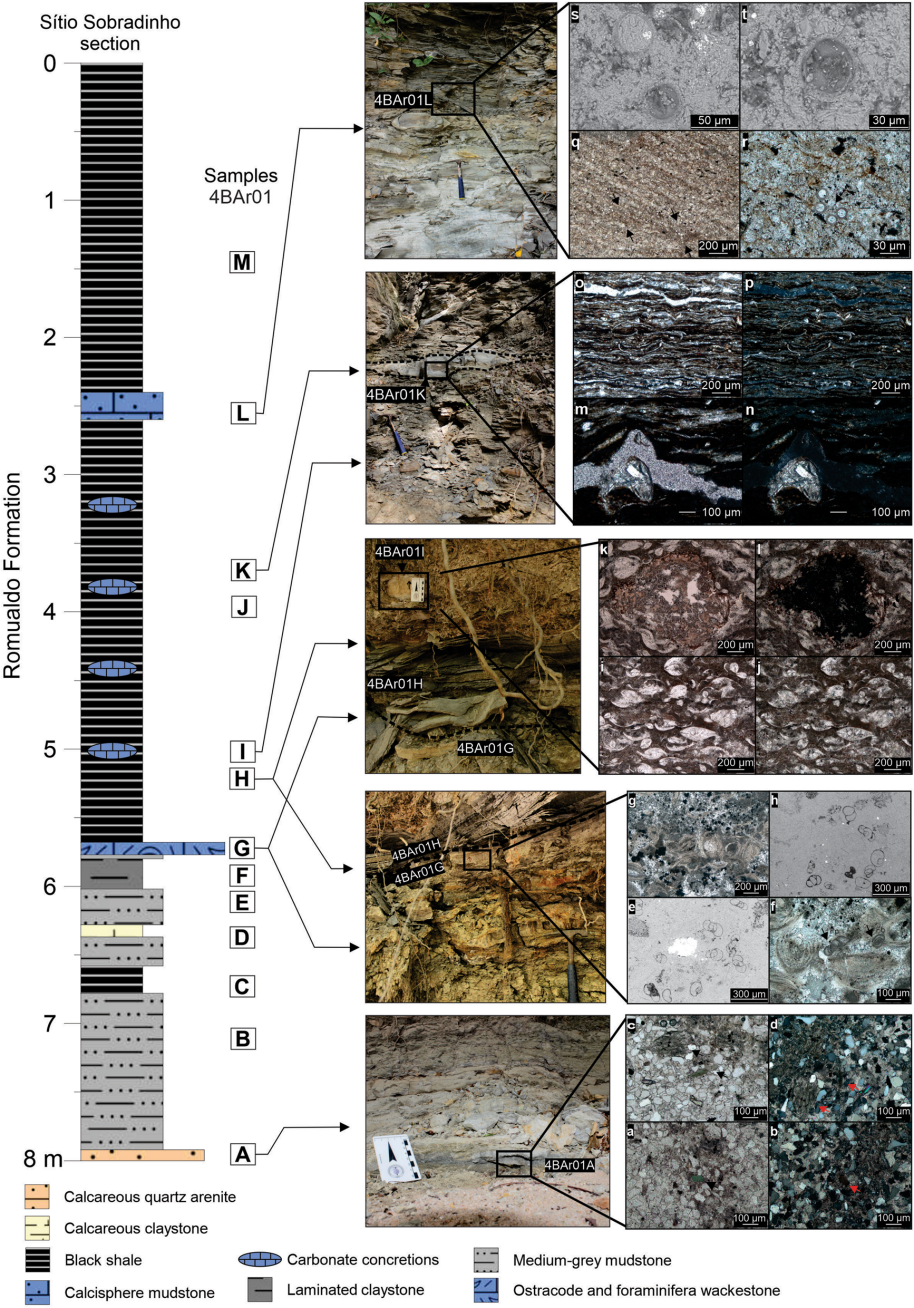
Lithological section of the interval studied here from the Sítio Sobradinho outcrop showing the positions of the stratigraphic samples (4BAr01A–4BAr01M) in the outcrop photographs and the facies photomicrographs to the right. The top of section (0 m) corresponds to the elevation datum 695 m. (**a**–**d**) Calcareous quartz arenite with glauconite grains (indicated by arrows): peloid type under PPL—parallel polarized light (**a**) and XPL—crossed polarized light (**b**) and foliated grains under PPL (**c**) and XPL (**d**). (**e**–**g**) Massive fossiliferous wackestone: foraminifera casts in SEM images (**e**, **h**), foraminifera, ostracode valves and peloids in a sparry calcite cement under XPL (**f**), non-oriented ostracode valves and peloids in a micritic matrix and several metallic grains (black euhedral and framboid) under PPL (**g**). (**i**–**l**) Laminated ostracode wackestone: oriented ostracode valves and carapaces in a micritic matrix under PPL (**i**) and XPL (**j**) and a silica grain disturbing the orientation of ostracodes under PPL (**k**) and XPL (**l**). (**m**–**p**) Carbonaceous and fossiliferous shale: an ostracode carapace disturbing the lamination under PPL (**m**) and XPL (**n**) and wavy carbonaceous and clay laminae with intercalated ostracode valves and few silty grains under PPL (**o**) and XPL (**p**). (**q**–**t**) Calcisphere mudstone: calcispheres indicated by arrows under PPL (**q**, **r**) and calcispheres in SEM images (**s**,** t**). Figure created by Juliana Guzmán on CorelDRAW version 22.0 (https://www.coreldraw.com).

The stratigraphic interval here studied is characterized by a general fining-upward gradation from fine-grained sandstone to siltstone, laminated claystone and an organic-rich shale succession with carbonate rocks (Fig. 2). From the base to the top, eight lithofacies were identified, and the microfacies of seven of these were analysed. Lithofacies A is a massive, fine-grained calcareous quartz arenite with chlorite, muscovite and pellets or foliated grains of glauconite (Fig. 2a–d). Lithofacies B corresponds to a massive medium-grey to light yellow siltstone (the same for sample 4BAr01E and calciferous sample 4BAr01D). Lithofacies F is a laminated and lenticular organic-rich claystone with pyrite. From a laterally continuous level, lithofacies G is defined as a massive wackestone with abundant ostracodes and foraminifera, pyrite grains are also observed (Fig. 2e–h). From a sample concretion, lithofacies I corresponds to a laminated ostracode wackestone with rare foraminifera and silica grains (Fig. 2i–l). Lithofacies K corresponds to a shale package containing interbeds, concretions and lenses of limestone, its microfacies is characterized by wavy carbonaceous and clay laminae with intercalated ostracode valves (Fig. 2m–p). Lithofacies L, which is a laterally discontinuous lens, corresponds to a calcisphere mudstone with rare foraminifera and ostracodes (Fig. 2q–t).

**Figure 3:**
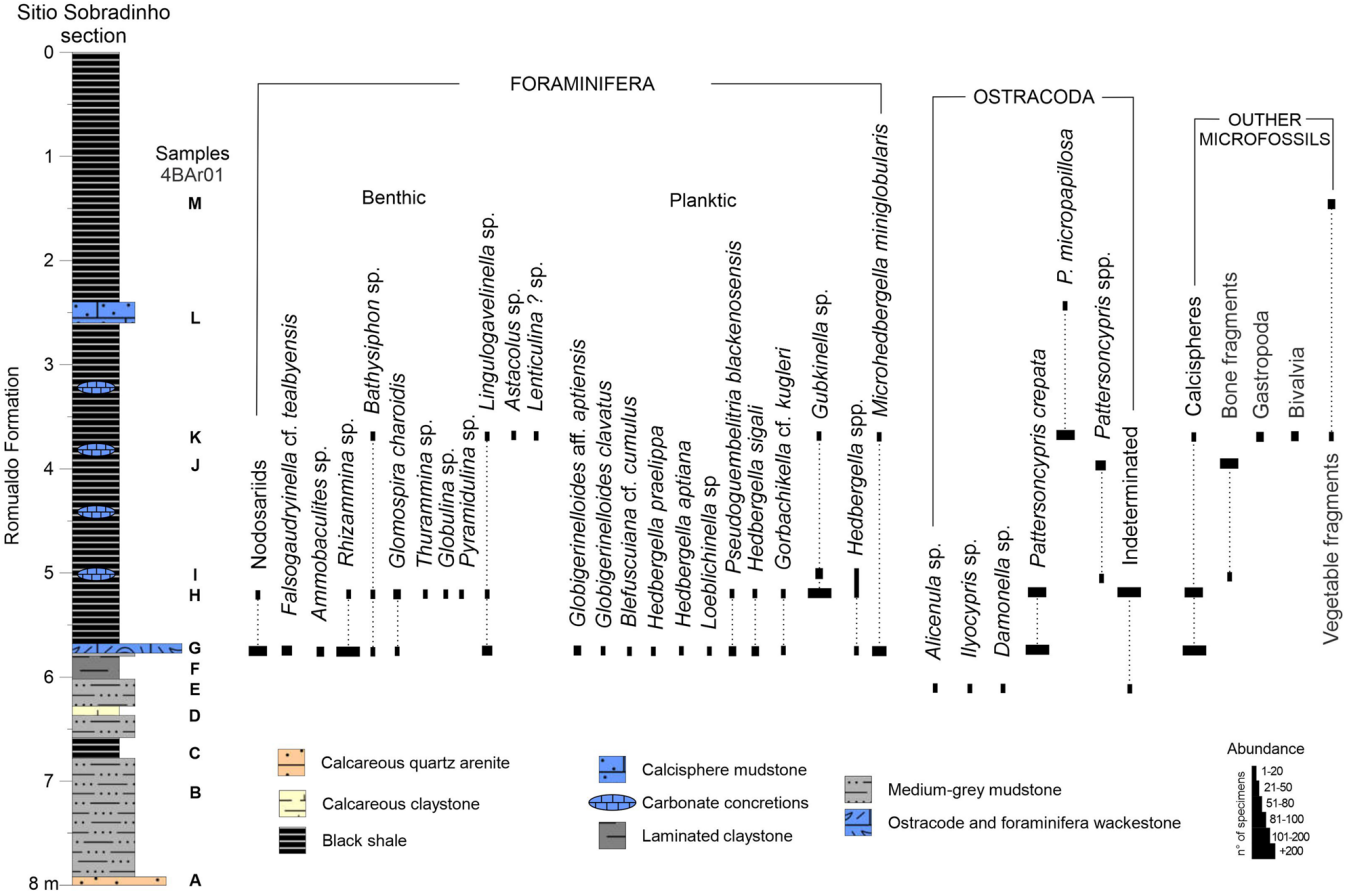
Distribution and abundance of microfossils recovered from the Sítio Sobradinho section. Figure created by Robbyson Mendes Melo on CorelDRAW version 22.0 (https://www.coreldraw.com).

The recovered microfossil assemblages are mainly represented by foraminifera, ostracodes and calcispheres, which have a constant presence in the middle portion (samples 4BAr01G–4BAr01L) of the section, with moderate diversity and an abundance of recovered specimens. The first samples corresponding to the base of the section are sterile, one possibility could had been due to dissolution during diagenesis. In the upper portion of the section, there is a decrease in the occurrence of ostracodes and foraminifera, and microgastropod specimens, bivalves, bone fragments and plant fragments (bryophyte capsules) are recovered. The distribution and abundance of the different taxonomic groups recovered are shown in Fig. 3.

**Figure 4:**
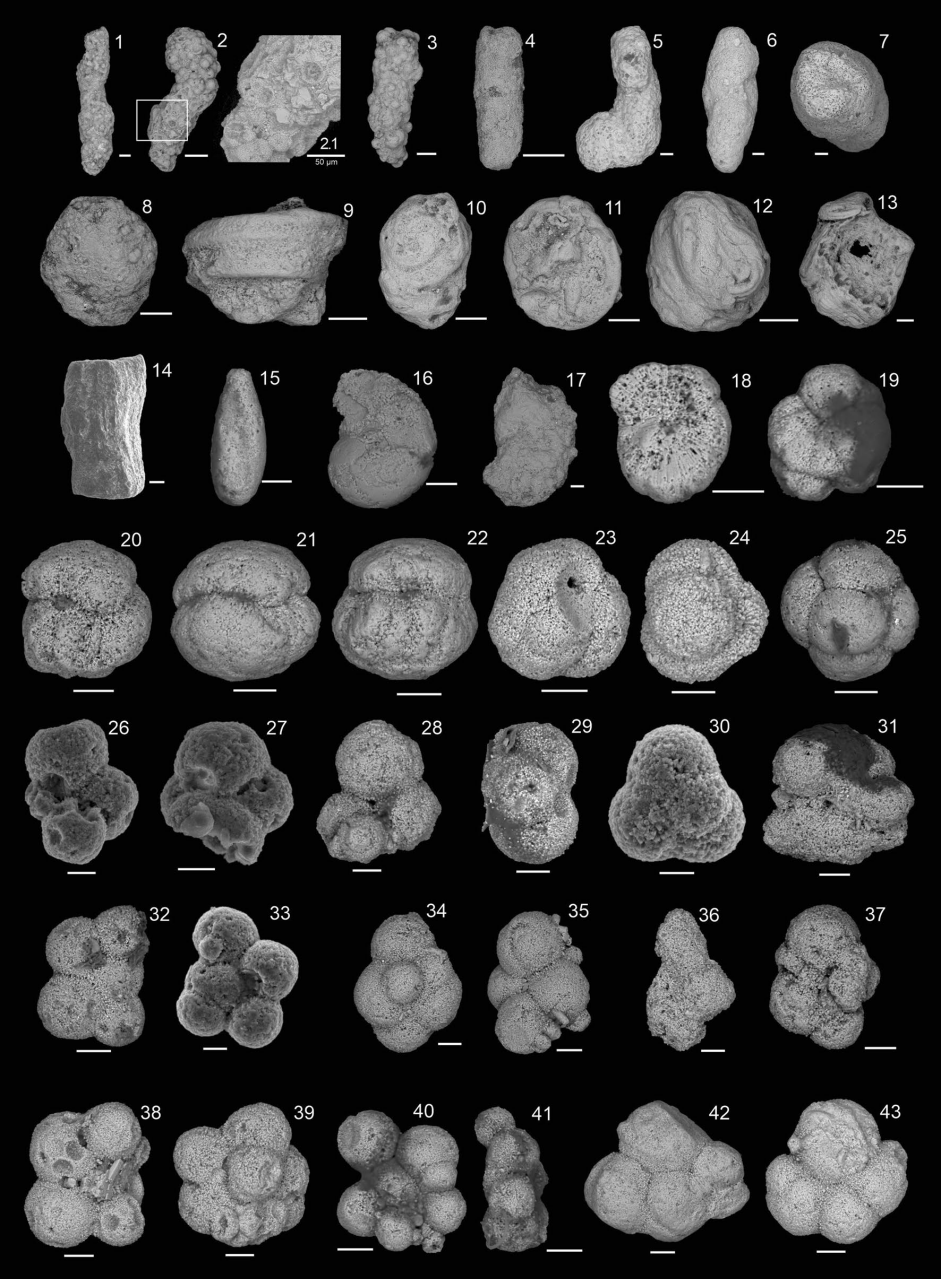
Foraminifera recovered from the Sítio Sobradinho section: 1–19. Benthic foraminifera: 1–3*. Rhizammina* sp., 1. LMA-00043, 2. LMA-00044, 3. LMA-00045, sample 4BAr01G; 4. *Bathysiphon* sp., LMA-0004, sample 4BAr01K; 5. *Ammobaculites* sp., LMA-00047 sample 4BAr01G; 6–7. *Falsogaudryinella* cf. *tealbyensis*, LMA-00048, sample 4BAr01G; 8. *Thurammina* sp., LMA-00049, sample 4BAr01HD; 9–13. *Glomospira charoides*, 9. LMA-00050, 10. LMA-00051, 11–12, LMA00052, 13. LMA-00053, sample 4BAr01H; 14. *Pyramidulina* sp., LMA-00054, sample 4BAr01H; 15. *Globulina* sp., LMA-00055, sample 4BAr01H; 16. *Lenticulina*? sp., LMA-00056, sample 4BAr01K; 17. *Astacolus* sp., LMA-00057, sample 4BAr01K; 18–19. *Lingulogavelinella* sp., 18. LMA-00058, 19. LMA-00059, sample 4BAr01K; 20–43. Planktic foraminifera: 20–22. *Gubkinella* sp., 20. LMA-00060, 21, LMA-00061, 22. LMA-00062, sample 4BAr01H; 23–25. *Pseudoguembelitria blakenosensis*, 23. LMA-00063, 24. LMA-00064, 25. LMA-00065, sample 4BAr01G; 26–27. *Gorbachikella* cf. *kugleri,* 26. LMA-00066, 27. LMA-00067, sample 4BAr01G; 28–30. *Microhedbergella miniglobularis*, 28. LMA-00068, 29–30. LMA-00069, sample 4BAr01G; 31, 37. *Hedbergella praelippa*, 31. LMA-00070, 37. LMA-00075, sample 4BAr01G; 32–33. *Hedbergella aptiana*, 32. LMA-00071, 33. LMA-00072, sample 4BAr01G; 34–35. *Hedbergella sigali*, LMA-00043, sample 4BAr01G; 36. *Globigerinelloides clavatus*, LMA-00074, sample 4BAr01G; 38–39. *Blesfucuiana* cf. *cumulus*, LMA-00076, sample 4BAr01G; 40–41. *Globigerinelloides* aff. *aptiensis*, LMA-00077, sample 4BAr01G; 42–43. *Loeblichella* sp., 42. LMA-00078, 43. LMA-00079, sample 4BAr01G. Scale bar: 1–14 = 100 µm; 15–43 = 30 µm.

The samples yielded 17 foraminifera taxa, nine of plankton habit and eight of benthic habit. The benthic foraminifera are mainly represented by agglutinated forms, particularly *Rhizammina* sp., *Bathysiphon* sp., *Glomospira charoide*s and *Falsogaudryinella* cf. *tealbyensis* (Fig. 4), in addition to rare calcareous-hyaline test specimens (gavelinelids and nodosarids). Planktic foraminifera are well represented by small and abundant hedbergellids (*Hedbergella aptiana, H. praelippa, H. sigali*, *Blesfucuiana* cf. *cumulus* and mainly *Microhedbergella miniglobularis*). An abundant and important record of *Gubkinella* sp. is also noted in the section, in addition to *Gorbachikella* cf. *kugleri, Pseudoguembelitria blakenosensis, Globigerinelloides clavatus*, *Globigerinelloides* aff. *aptiensis*, and *Loeblichella* sp. (Fig. 4).

**Figure 5:**
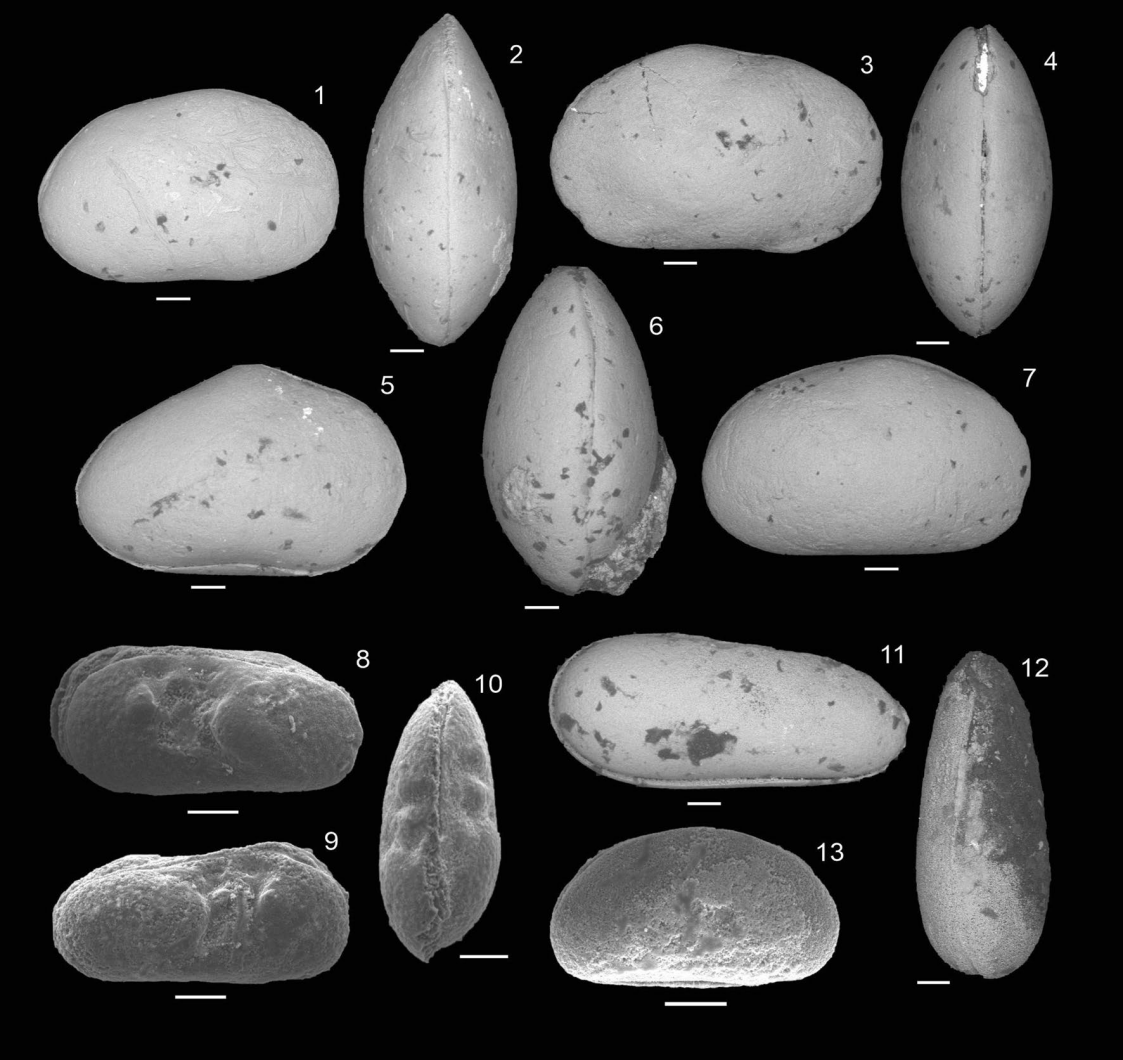
Ostracoda recovered from the Sítio Sobradinho section: 1–4, 7. *Pattersoncypris crepata*, 1. RV, LMA-00029, 2. DV, LMA-00030, 3. LV, LMA-00031, 4. DV, LMA-00032, 7. RV, LMA-00033, sample 4BAr01G; 5–6. *Pattersoncypris micropapillosa*, 5. RV, LMA-00034, 6. DV, LMA-00035, sample 4BAr01L; 8–10. *Ilyocypris* sp., 8. LV, LMA-00036, 9. DV, LMA-00037, 10. RV, LMA-00038, sample 4BAr01E; 11–12. *Alicenula* sp., 11. RV, LMA-00039, 12. DV, LMA-00040, sample 4BAr01E; 13. *Damonella* sp., RV, LMA00041, sample 4BAr01E. *RV* right view, *DV* dorsal view, *LV* left view. Scale bar: 100 µm.

From the ostracode fauna (Fig. 5), it is possible to recognize three associations: the first is represented by representatives of the genera *Alicenula, Ilyocypris* and *Damonella,* being registered below the first occurrence of foraminifera; the second association is composed of an abundant and monospecific association of *Pattersoncypris crepata* with more than 2000 specimens, associated with an abundant and diverse assemblage of benthic and planktic foraminifera; the third ostracode association is composed of *Pattersoncypris micropapillosa* and juvenile specimens of *Pattersoncypris* spp., which occur in the uppermost portion of the studied section.

## Discussion

**Figure 6:**
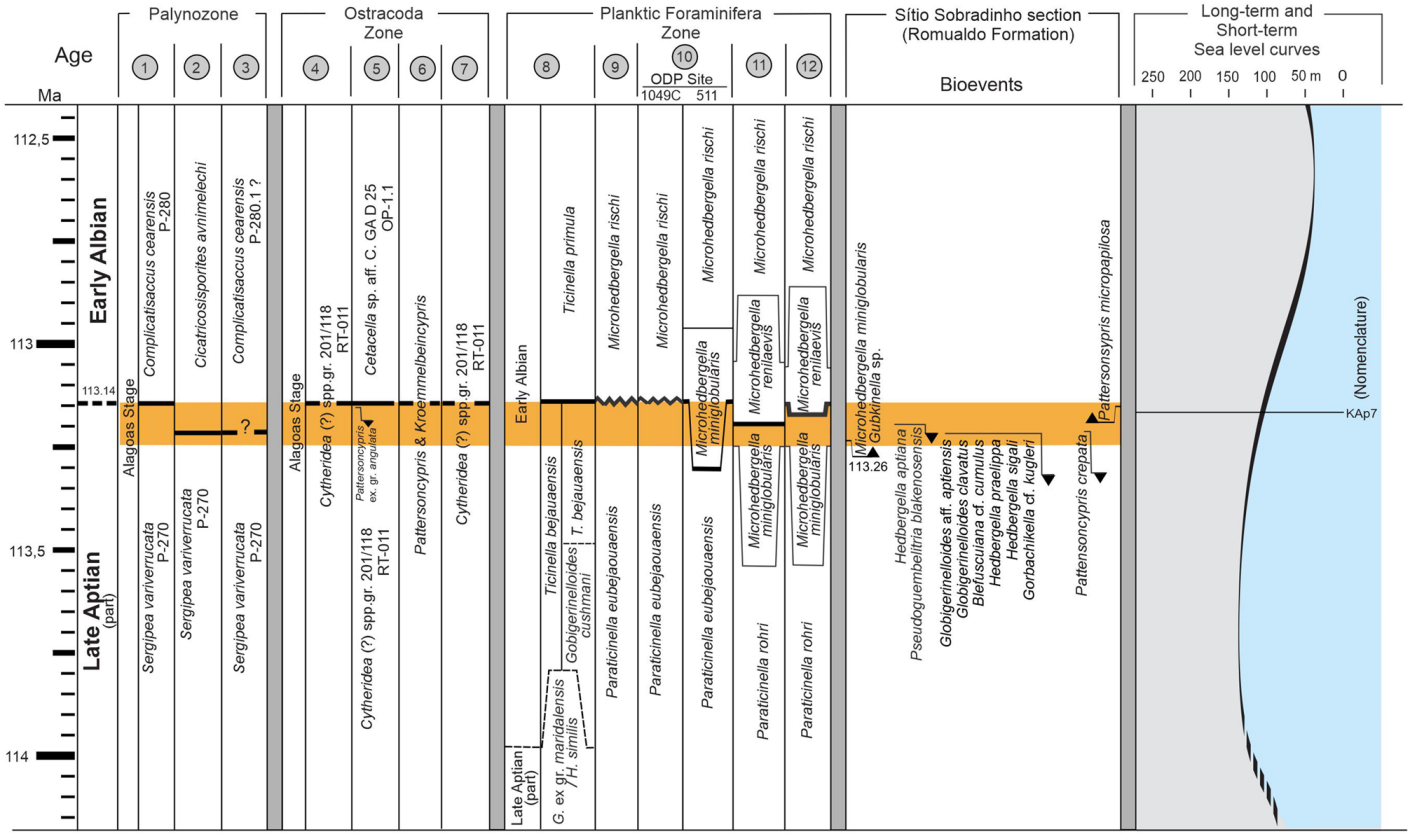
Correlation of planktic foraminifera, ostracodes and palynological zonal schemes proposed for the Aptian–Albian at global and local scales. Bioevents considered for the chronostratigraphic positioning of the studied interval in Sítio Sobradinho section (Araripe Basin). The orange rectangle delineates the studied section. Palynozone: 1. Sergipe-Alagoas Basin^67^; 2. integrated framework^116^; 3. Araripe Basin^68^; Ostracoda Zone: 4. Sergipe-Alagoas Basin^117^; 5. Potiguar Basin^118^; 6. integrated framework^119^; 7. Araripe Basin^6^; Planktic Foraminifera: 8. Sergipe Basin^78^; 9. DSDP Site 364^57^; 10. Umbria-Mache Basin^109^; 11. ODP Site 1049C e 511^50^; 12. Vocontian Basin^51^. The absolute ages follow the time scale of Ogg et al.^120^, TSCreator V7.4 and https://www.mikrotax.org/. Short-term (3rd order) sea level curves according to Haq^29^. Figure created by Robbyson Mendes Melo on CorelDRAW version 22.0 (https://www.coreldraw.com).

The late Aptian–early Albian biotic turnover records a dramatic extinction event of large-sized Aptian planktic foraminifera taxa and the appearance of few new small-sized Albian species. Based on planktic foraminifera, the late Aptian–early Albian boundary is marked by the last occurrences of typically Aptian taxa, particularly *Paraticinella rohri*^36,52–55^ (Fig. 6). The *Microhedbergella miniglobularis* Zone occurs above the extinction of *Paraticinella rohri* species*,* co-occurring with the last long-ranging Aptian hedbergellids and the first occurrence of *Microhedbergella renilaevis*^50^, in addition to the complete absence of planktic foraminifera in the > 250 μm size fraction^51^. The *Microhedbergella renilaevis* Zone corresponds to the biostratigraphic interval from the first occurrence of *Microhedbergella renilaevis* to the first occurrence of *Microhedbergella rischi*.

The sedimentary succession from Sítio Sobradinho is assigned to the upper Aptian. The recovered association is composed of *Hedbergella aptiana*, *H. praelippa, H. sigali*, *Blesfucuiana* cf. *cumulus*, *Gorbachikella* cf. *kugleri*, *Pseudoguembelitria blakenosensis* and *Microhedbergella miniglobularis,* which allows a chronostratigraphic position in the upper Aptian, correlated with international foraminiferal zonal schemes^50,51,55–57^. In addition, the co-occurrence of *Pattersoncypris crepata*, whose record is very characteristic of the middle–upper Aptian from Brazilian deposits, in the Araripe Basin^58^, Potiguar Basin^59^ and Sergipe-Alagoas Basin^60^ is correlated with a palynological zone coded as P-270^59^ and corroborates the foraminifera dating. These data reinforce the potential of *Pattersoncypris crepata* for the dating of Aptian strata. The top of the section contains *Microhedbergella miniglobularis* and *Gubkinella* sp. associated with *Pattersoncypris micropapillosa*, the last of which is an ostracode species recorded in the Aptian–Albian interval^3,43,46,49,61–65^. Moreover, typical Albian assemblage not recovered from our samples (e.g., *Microhedbergella renilaevis* and *Microhedbergella rischi*), that marks the top of the *Microhedbergella miniglobularis* Zone^50^ and the base of the Albian, corroborates the Aptian age for the section. According to Petrizzo et al.^51^, the first occurrence of *Microhedbergella renilaevis* marks an important bioevent that represents a major step in the evolution and diversification of the Albian planktic fauna, an event that was not identified in the studied section. Furthermore, the chronostratigraphic positioning of the lithological section studied in the upper Aptian, can be corroborated by the occurrence of *Sergipea variverrucata* Palynozone P-270^66–68^, which was identified throughout the Sítio Sobradinho section by Teixeira et al.^69^.

The aspects involving the age and direction of marine ingression into the Araripe Basin and northern South Atlantic Ocean have been the subject of active discussion for many years^24–27^, as illustrated in Fig. 1. In northeast Brazil, the Aptian transgression was sufficiently extensive that evaporites were deposited even in the interior basins. In the Araripe Basin, this transgression is represented by the Ipubi Formation and the typical marine fossil record towards the top of the Romualdo Formation. Based on the fossils, several routes have been hypothesized for the marine ingression into the Araripe Basin, that is, through the Parnaíba, Sergipe or Potiguar Brazilian basins, as a junction of three seaways that effectively connected these basins or (as a highly speculative hypothesis) via an extensive seaway from northwestern South America^25,26^. On the other hand, considering only stratigraphic and sedimentologic information, Assine et al.^27^ suggested that the Parnaíba and Potiguar basins were set apart from each other and from the Araripe Basin, configuring marine transgression towards these respective basins from the north, northeast and southeast. This hypothesis and the available information on the fluvial southeast-directed palaeocurrents of the Barbalha Formation (the base of the Santana Group) flowing towards the Jatobá, Tucano and Recôncavo rift system indicates continental palaeodrainage^70^ and reinforce the existence of an epeiric sea following upstream river valleys into the Araripe Basin from the southeast^23^. Nevertheless, the present structural framework of the Araripe Basin corresponds to a graben inverted into a high-standing horst due to the stress field imposed by ridge-push forces from the Mid-Atlantic Ridge to the west and from the Andes to the east mainly during the Quaternary, and rift-related normal faults concentrated along inherited shear zones were reactivated to form the main inversion faults^71^. The aforementioned palaeocurrents that exist along the generally inverted western margin of South America may be altered from their original orientations, and thus, interpretations of Cretaceous flowing directions based on these palaeocurrents should be re-evaluated. However, palaeobiogeographical differentiation is observed in the Aptian microfossil association of the South Atlantic as a result of the physical barrier in the Santos–Benguela segment that hinders the free circulation and mixing of seawater between the southern and central to equatorial South Atlantic^24^. In relation to foraminifera, the absence of several species of tropical/subtropical planktic foraminifera from the Aptian–Albian sedimentary successions in the southernmost sector of the northern South Atlantic Ocean (north of the Walvis Ridge-Rio Grande Rise), resulted in tentative zonal assignments based on the occurring assemblages, and this pattern has been interpreted as an effect of a possible Austral palaeobiogeographical affinity^72–74^. The studies of Koutsoukos^56^ and Kochhann et al.^57^, which addressed the foraminiferal assemblages of the Sergipe Basin (Brazil) and DSDP Site 364 (offshore Angola), indicate a tropical/subtropical affinity for several species, suggesting that these areas had at least surface-water exchanges with the western Tethyan biogeographic provinces of the low-latitude central North Atlantic, possibly even at intermediate (epi- to mesopelagic) water depths. The last referred authors reinforced the theory of a surface-water connection between the proto-central Atlantic Ocean and the southernmost sector of the northern South Atlantic Ocean (north of the Walvis Ridge-Rio Grande Rise) during the late Aptian.

The Aptian foraminiferal association presented here has a tropical/subtropical affinity when compared with previously described associations (Sabinas Basin, Mexico^75^; DSDP Leg 79, offshore central Morocco^76^; Sergipe Basin, Brazil^56,77,78^; Cassis-La Bédoule, France^36,79^; DSDP Leg 40, Sites 363–364, Angola Basin and Walvis Ridge, offshore Angola^57,73^; Vocontian Basin, Itália^51^). The genera *Gorbachikella*, *Blesfucuiana* and *Pseudoguembelitria* have a very restricted latitudinal range, only in North Africa, Eastern Europe, Central America (including the Caribbean and Mexico) and the subtropical western North Atlantic^50,75,80–84^. Biogeographic evidence suggests that these globular forms can be considered indicators of warm sea surface temperatures, the occurrence of which is related to the emergence of possibly eutrophic and/or climatic (probably hot) oceanographic conditions^83,85,86^. Thus, the data presented herein support a surface-water marine connection with open-marine and shallow-water foraminiferal assemblages^38^ between the central Atlantic Ocean by the late Aptian. This connection could be related to the global sea-level rise reported at that time^24,29,56,77,87–89^.

**Figure 7:**
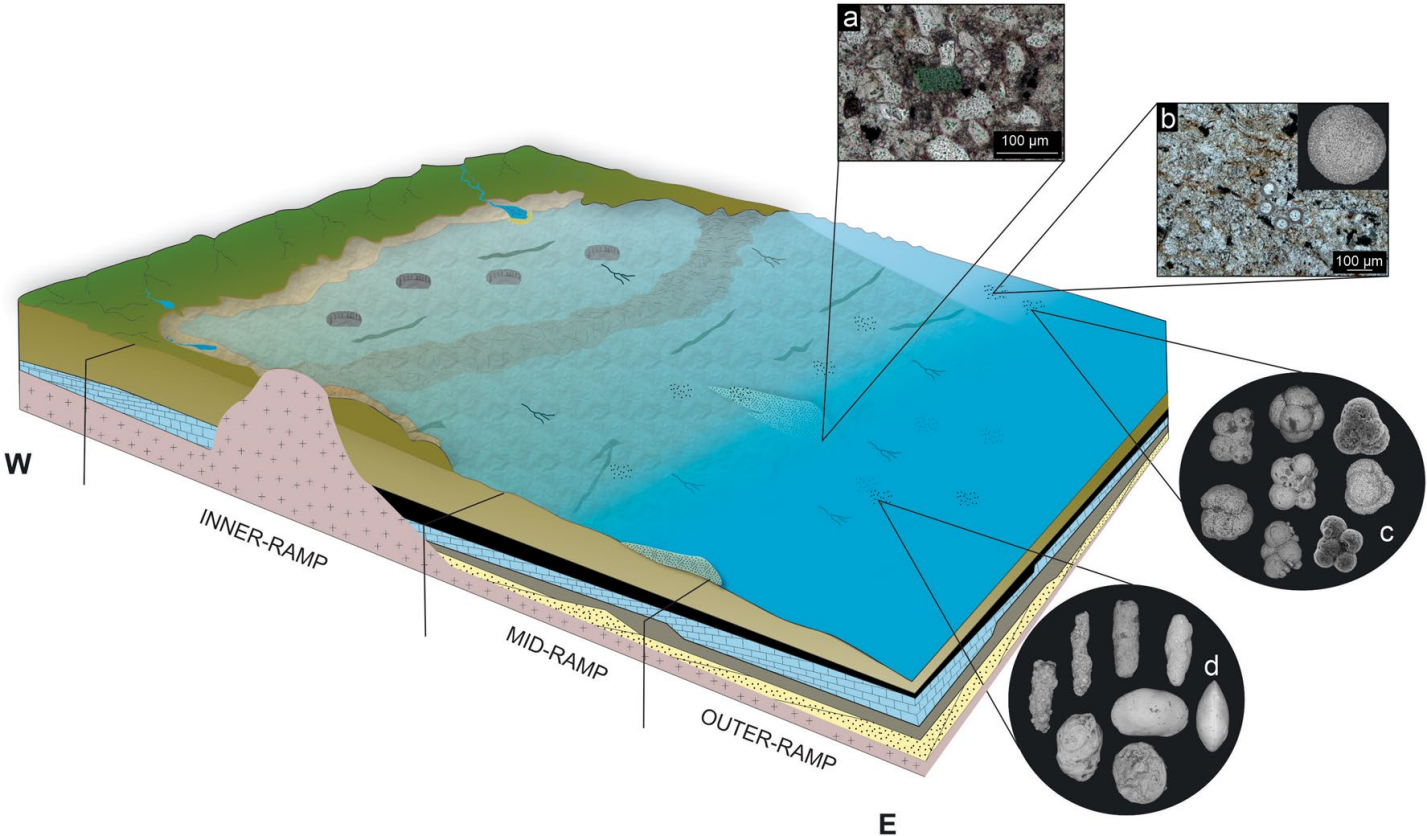
Schematic palaeoenvironmental reconstitution of the Romualdo Formation deposits: (**a**) Glauconite-bearing sandstone shoal; (**b**–**d**) Shale interbedded with ostracode and foraminifera wackestone and calcisphere mudstone: (**b**) calcispheres; (**c**) planktic foraminifera; (**d**) benthic foraminifera and ostracode *Pattersoncypris crepata*. The interpretation of the west portion of the Araripe Basin is based on Varejão et al.^90^ data. The complete figure was composed by Robbyson Melo on CorelDRAW version 22.0 (https://www.coreldraw.com).

The eight meters interval studied here corresponds to the maximum flooding zone identified by Custódio et al.^23^ in the total ~ 100 m of the Romualdo Formation at the Sítio Sobradinho outcrop. The vertical distributions of the lithological and palaeontological macro- and microfacies in the stratigraphic interval result in the division into two facies associations related to a deepening-upward sequence on a ramp-type and mixed siliciclastic-carbonate marine shelf (Fig. 7). To the western of the Araripe Basin, Varejão et al.^90^ described microbialites and stromatolites associated to rocky-protected lagoon, that corroborates our interpretation of a low-gradient ramp, deeper to the east of the basin where Sítio Sobradinho Section is located. The lower studied interval corresponds to the mid-ramp shoal facies association, which groups the glauconite-bearing calcareous quartz arenite and the siltstone to claystone overlaid strata. From this interval, rare freshwater and transitional ostracode occurrences, represented by the genera *Damonella, Alicenula* and *Ilyocypris*^46,91–96^, were recorded in samples below the first occurrences of foraminifera, suggesting the allochthonous origin of these ostracofauna. In addition, the marine phase in the basin is reinforced by the presence of glauconite as foliated and peloidal grains, which represent the seafloor synsedimentary glauconitization of detrital biotites and a diagenetic product, respectively, which formed especially during intervals of mildly reducing conditions^97,98^. Glauconite formation is a slow process that requires low rates of sediment accumulation to allow the long-term contact of detrital grains with seawater in primarily mid- to outer-shelf settings^97^. According to Flügel^99^, syndepositional authigenic glauconite indicates a break in sedimentation, commonly in deep subtidal and bathyal environments. The greatest period of glauconite formation occurred in the Cretaceous, notably along the continental margins of the widening North Atlantic Ocean, when the global sea level was high^100^.

The upper studied interval corresponds to the outer ramp to basin facies association and is characterized by a carbonaceous and fossiliferous shale package interbedded by plankton-bearing wackestone and mudstone as continuous beds, lenses and concretions. A depositional environment with palaeodepths estimated between fifty and two hundred metres can be corroborated by the dominance of benthic foraminifera with a tubular form (the M1 morphogroup), infaunal organisms (*Rhizammina* and *Bathysiphon*), and less abundant but still frequent reophacellids (*Falsogaudryinella*) and ammodiscids (*Glomospira*), which indicate poorly oxygenated conditions in a middle neritic-upper bathyal environment represented by suspension feeders that prefer a low organic carbon flux and high dissolved oxygen content^101–104^. The lack of a continuous reef and the lack of shallow water-derived clasts in deeper-water sediments led to the characterization of the Sítio Sobradinho section as having been deposited on a ramp-type marine shelf without a steep slope at the edge (e.g., Flügel^99^). In addition, the presence of a monospecific fauna, *Pattersoncypris crepata*, associated with agglutinated foraminifers indicates its tolerance to higher-salinity conditions, and thus, it is considered a holoeuryhaline species, as was interpreted from its record with the first marine incursion in the Potiguar Basin^59,105–107^. Furthermore, in the Sergipe-Alagoas Basin, the species *Pattersoncypris crepata* occurs at the same levels as the typical marine ostracodes of the genera *Cytherella, Cytherelloidea*, *Patellacythere* and *Aracajuia*^60^.

In the eastern part of the Araripe Basin, the Romualdo depositional sequence records a transgressive–regressive cycle comprising a transgressive system tract with tide-dominated coastal facies to outer-shelf black shales as the maximum flooding zone and a highstand system tract characterized by a progradational package that records gradual continentalization^23^. The two facies associations we recognized macro- and microscopically can be correlated with the inner to outer shelf, dysoxic to anoxic facies association identified by the previously mentioned authors with the important difference that no proximal facies were observed in the interval analysed herein. The textural framework and the carbonate grains (‘allochems’) determined in the studied carbonate microfacies indicate that the carbonate rocks associated with the organic-rich shale succession represent the 2 – 5 ramp microfacies types (RMFs) (sensu Flügel^99^), so it is assumed that the studied interval was deposited from the mid-ramp shoal to basin positions. This palaeoenvironmental interpretation is reinforced by the distribution of the identified foraminifera and ostracode microfossils.

The low diversity-assemblage of planktic foraminifera recovered from the Sítio Sobradinho section is predominantly composed of small hedbergellids (*Hedbergella* and *Microhedbergella*) and chilostomellids (*Gubkinella*), which are characteristic of late Aptian–early Albian deposits around the world^51,56,57,73,76,108^, whose deposition may be related to OAE 1b^35,109,110^. The post-*Paraticinella rohri* ecosystem promoted the proliferation of very small planktic foraminiferal taxa (i.e., microhedbergellids) and siliceous plankton (i.e., radiolarian), which thrived as opportunistic/disaster taxons^109,110^. According to Sabatino et al.^111^, the ecological behaviour of *M. miniglobularis,* whose record in the analysed section is abundant*,* seems to be that of a disaster opportunist since it occurred and thrived during a period of high but variable environmental stresses when no other planktic foraminifera are present, whereas it is rare or absent in normal environments. In addition, the abundant record of *Gubkinella* sp. supports the idea of a restricted environment after a rapid connection with the sea since representatives of this genus are generally less abundant in open-ocean environments and probably have broad ecological (eurytopic) tolerances, being especially characteristic of neritic environments, continental margins, epicontinental seas^75,108,112^ and areas of upwelling^113^. According to Leckie^113,114^, the genus *Gubkinella* is one of the main representatives of the “Epicontinental Sea Fauna”, composing an average of 4.5% of the assemblage. The variable abundance of *Gubkinella* (epicontinental plankton) relative to *Hedbergella* and *Globigerinelloides* (open-marine shallow-water plankton) suggests a relatively shallow continental margin and/or highly fertile surface waters (possibly within 500 m) in a restricted environment at the time of deposition based on the palaeoecological model of Leckie^113^. Moreover, abundances of these “Epicontinental Sea Fauna”, described in the upper Aptian of northwest Africa^76^ and along the Antarctic margin^114^, similar as we recorded, are perhaps associated with highly productive continental margins^113^. The sedimentary organic matter content of the Sítio Sobradinho section^69^ suggests a position more distal to intermediate in the context of the epicontinental shallow sea. At the top of the section, the decreased occurrence of foraminifera, associated with the other groups (bivalves, gastropods, plant fragments) and especially *P. micropapillosa*, may indicate a change in environmental conditions, with the beginning of a mixohaline to limnic environment^6,43,63^ that can characterize very proximal conditions but with the high influence of components of marine origin, evidencing a proximal–distal transitional coastal marine environment^69^.

In summary, this integrated study of foraminifera, ostracodes and other microfossil data from the Romualdo Formation indicates that the local Alagoas Stage (Ostracoda Zone RT-011) can now be constrained to the Aptian. The foraminiferal assemblages show Tethyan affinities suggesting a marine route coming to the Araripe Basin from the equatorial South Atlantic with north seawater origin.

## Supplementary information

Supplementary file 1

## References

[1] Maisey, J. G. *Santana Fossils: an illustrated atlas*. (T.F.H Publications, Inc., 1991).

[2] Martill DM (2007). The age of the Cretaceous Santana Formation fossil Konservat Lagerstätte of north-east Brazil: a historical review and an appraisal of the biochronostratigraphic utility of its palaeobiota. Cretac. Res..

[3] Bate RH (1972). Phosphatized ostracod with appendages from the Lower Cretaceous of Brazil. Paleontology.

[4] Price LI (1973). Quelônio Amphichelydia no Cretáceo Inferior do Nordeste do Brasil. Rev. Bras. Geociências.

[5] Silva M, Arruda G (1976). Insetos (Hymenoptera) cretáceos do Grupo Araripe - Nordeste do Brasil. An. do Inst. Ciências Biológicas.

[6] Coimbra JC, Arai M, Carreño AL (2002). Biostratigraphy of Lower Cretaceous microfossils from the Araripe Basin, northeastern Brazil. Geobios.

[7] Kellner, A. W. A. & Campos, D. de A. The function of the cranial crest and jaws of a unique pterosaur from the Early Cretaceous of Brazil. *Science (80-).* **297**, 389–392 (2002). 10.1126/science.1073186 12130783

[8] Mohr, B. A. R., Bernardes-de-Oliveira, M. E. C. & Loveridge, R. F. The macrophyte flora of the Crato Formation. *Crato Foss. Beds Brazil Wind. into an Anc. World* 537–565 (2007) 10.1017/CBO9780511535512.020.

[9] De Lima FJ, Saraiva AAF, Sayão JM (2012). Revisão da paleoflora das formações Missão Velha, Crato e Romualdo, Bacia do Araripe Nordeste do Brasil. Estud. Geológicos.

[10] Salisbury SW, Frey E, Martill DM, Bucchy MC (2003). A new mesosuchian crocodilian from the Lower Cretaceous Crato Formation of North-Eastern Brazil. Palaeontographica.

[11] Brito, P. M. & Yabumoto, Y. An updated review of the fish faunas from the Crato and Santana formations in Brazil, a close relationship to the Tethys fauna. *Bull. Kitakyushu Museum Nat. Hist. Hum. Hist. Ser. A* **9**, 107–136 (2011).

[12] Arai M, Neto JB, Lana CC, Pedrão E (2000). Cretaceous dinoflagellate provincialism in Brazilian marginal basins. Cretac. Res..

[13] Prado, L. A. C. do, Fambrini, G. L. & Barreto, A. M. F. Tafonomy of macroinvertebrates and Albian marine ingression as recorded by the Romualdo Formation (Cretaceous, Araripe Basin, Brazil). *Brazilian J. Geol.* **48**, 519–531 (2018).

[14] Prado LAC, Luque J, Barreto AMF, Palmer AR (2018). New brachyuran crabs from the Aptian-Albian Romualdo Formation, Santana Group of Brazil: Evidence for a Tethyan connection to the Araripe Basin. Acta Palaeontol. Pol..

[15] Prado, L. A. C., Calado, T. C. dos S. & Barreto, A. M. F. New records of shrimps from the Lower Cretaceous Romualdo Formation, Araripe Basin, northeastern Brazil, with new taxa of Penaeoidea (Crustacea: Decapoda: Dendrobranchiata). *Cretac. Res.* **99**, 96–103 (2019).

[16] Fambrini GL (2011). Estratigrafia, arquitetura deposicional e faciologia da Formação Missão Velha (Neojurássico-Eocretáceo) na área-tipo, Bacia do Araripe, Nordeste do Brasil: Exemplo de sedimentação de estágio de início de rifte a clímax de rifte. Geol. USP - Ser. Cient..

[17] Assine ML (2007). Bacia do Araripe. Bol. Geociências da Petrobras.

[18] Brito Neves, B. B., Santos, E. J. & Van Schmus, W. R. Tectonic history of the Borborema Province. in *Tectonic Evolution of South America* (eds. Cordani, U., Milani, E., Thomaz Filho, A. & Campos, D. A.) 151–182 (2000).

[19] Cesero, P. & Ponte, F. C. Análise comparativa da paleogeologia dos litorais atlânticos brasileiro e africano. *Bol. Geociências da Petrobras* **11**, 1–18. 1997 (1972).

[20] Kuchle, J., Scherer, C. M. dos S., Born, C. C., Alvarenga, R. dos S. & Adegas, F. A contribution to regional stratigraphic correlations of the Afro-Brazilian depression - The Dom João Stage (Brotas Group and equivalent units - Late Jurassic) in Northeastern Brazilian sedimentary basins. *J. South Am. Earth Sci.* **31**, 358–371 (2011).

[21] Scherer, C. M. dos S. *et al.* Tectono-stratigraphic evolution of the Upper Jurassic-Neocomian rift succession, Araripe Basin, Northeast Brazil. *J. South Am. Earth Sci.* **49**, 106–122 (2014).

[22] Assine ML (2014). Sequências deposicionais do Andar Alagoas da Bacia do Araripe, Nordeste do Brasil. Bol. Geociências da Petrobras.

[23] Custódio MA (2017). The transgressive-regressive cycle of the Romualdo Formation (Araripe Basin): Sedimentary archive of the Early Cretaceous marine ingression in the interior of Northeast Brazil. Sediment. Geol..

[24] Arai M (2009). Paleogeografia do Atlântico Sul no Aptiano: um novo modelo a partir de dados micropaleontológicos recentes. Bol. Geociencias da Petrobras.

[25] Arai M (2014). Aptian/Albian (Early Cretaceous) paleogeography of the South Atlantic: a paleontological perspective. Braz. J. Geol..

[26] Arai, M. Reply to the comments of Assine et al. (Comments on paper by M. Arai ‘Aptian/Albian (Early Cretaceous) paleogeography of the South Atlantic: A paleontological perspective’). *Braz. J. Geol.* **46**, 9–13 (2016).

[27] Assine, M. L., Quaglio, F., Warren, L. V. & Simões, M. G. Comments on paper by M. Arai ‘Aptian/Albian (Early Cretaceous) paleogeography of the South Atlantic: A paleontological perspective’. *Braz. J. Geol.* **46**, 3–7 (2016).

[28] Heine C, Zoethout J, Müller RD (2013). Kinematics of the South Atlantic rift. Solid Earth.

[29] Haq BU (2014). Cretaceous eustasy revisited. Glob. Planet. Change.

[30] Jenkyns, H. C. Carbon-isotope stratigraphy and paleoceanographic significance of the Lower Cretaceous shallow-water carbonates of Resolution Guyot, Mid-Pacific Mountains. *Proc. Ocean Drill. Program, 143 Sci. Results* (1995) 10.2973/odp.proc.sr.143.213.1995.

[31] Jenkyns, H. C. Geochemistry of oceanic anoxic events. *Geochemistry, Geophys. Geosystems* **11**, 1–30 (2010).

[32] Premoli Silva, I., Erba, E., Salvini, G., Locatelli, C. & Verga, D. Biotic changes in Cretaceous oceanic anoxic events of the Tethys. *J. Foraminifer. Res.* **29**, 352–370 (1999).

[33] Kuhnt W, Holbourn A, Moullade M (2011). Transient global cooling at the onset of early Aptian oceanic anoxic event (OAE) 1a. Geology.

[34] Bottini C (2015). Climate variability and ocean fertility during the Aptian Stage. Clim. Past.

[35] Leckie RM (1989). A paleoceanographic model for the early evolutionary history of planktonic Foraminifera. Palaeogeogr. Palaeoclimatol. Palaeoecol..

[36] Premoli Silva I, Sliter WV (1999). Cretaceous paleoceanography: Evidence from planktonic foraminiferal evolution. Spec. Pap. Geol. Soc. Am..

[37] Kennedy WJ (2000). Integrated stratigraphy across the Aptian-Albian boundary in the Marnes Bleues, at the Col de Pre-Guittard, Arnayon (Drome), and at Tartonne (Alpes-de-Haute-Provence), France: a candidate global boundary stratotype section and boundary point for the base. Cretac. Res..

[38] Leckie, R. M., Bralower, T. J. & Cashman, R. Oceanic anoxic events and plankton evolution: Biotic response to tectonic forcing during the mid-Cretaceous. *Paleoceanography* **17**, 13–1–13–29 (2002).

[39] Berthou, P., Viana, M. S. & Campos, D. Coupe de la Formation Santana dans le secteur de Pedra Branca (Santana do Cariri; Bassin d’Araripe, NE du Brésil): contribution a l’étude de la sedimentologie et des paleoenvironnements. in *Simpósio sobre a Bacia do Araripe e Bacias Interiores do Nordeste, 1* 173–191 (URCA, 1990).

[40] Araripe R (2019). Análise isotópica de oxigênio e carbono em microfósseis da Formação Romualdo, Cretáceo Inferior, Bacia do Araripe, Pernambuco Nordeste do Brasil. Geochim. Bras..

[41] Carvalho, S.G. & Carvalho, M. F. Microfácies sedimentares do furo GSB-1, Serra Branca, chapada do Araripe, Pernambuco. *Estud. Sedimentológicos [UFRN]* **3/4**, 49–63 (1974).

[42] Lima MR (1979). Paleontologia da Formação Santana (Cretáceo do Nordeste do Brasil): estágio atual do conhecimento. An. Acad. Bras. Cienc..

[43] Arai, M. & Coimbra, J. C. Análise paleoecológica do registro das primeiras ingressões marinhas na Formação Santana (Cretáceo Inferior da Chapada do Araripe). in *1° Simpósio Sobre a Bacia do Araripe e Bacias Interiores do Nordeste* 226–233 (1990).

[44] Goldberg K, Premaor E, Bardola T, Souza PA (2019). Aptian marine ingression in the Araripe Basin: Implications for paleogeographic reconstruction and evaporite accumulation. Mar. Pet. Geol..

[45] Berthou P, Depeche F, Colin J, Figueira JB, Teles MS (1994). New data on the ostracodes from the Crato lithologic unit (lower member of the Santana Formation, latest Aptian–lower Albian) of the Araripe Basin (Northeastern Brazil). Acta Geológica Leopoldensia.

[46] Tomé, M. E. T. R., Lima Filho, M. F. & Neumann, V. H. M. L. Taxonomic studies of non-marine ostracods in the Lower Cretaceous (Aptian-lower Albian) of post-rift sequence from Jatobá and Araripe basins (Northeast Brazil): Stratigraphic implications. *Cretac. Res.* **48**, 153–176 (2014).

[47] Bate RH (1971). Phosphatized ostracods from the Cretaceous of Brazil. Nature.

[48] Smith RJ (1999). Possible fossil ostracod (Crustacea) egg from the Cretaceous of Brazil. J. Micropaleontol..

[49] Smith RJ (2000). Morphology and ontogeny of Cretaceous ostracods with preserved appendages from Brazil. Palaeontology.

[50] Huber BT, Leckie RM (2011). Planktic foraminiferal species turnover across deep-sea Aptian/Albian boundary sections. J. Foraminifer. Res..

[51] Petrizzo MR, Huber BT, Gale AS, Barchetta A, Jenkyns HC (2012). Abrupt planktic foraminiferal turnover across the Niveau Kilian at Col de Pré-Guittard (Vocontian Basin, southeast France): New criteria for defining the Aptian/Albian boundary. Newsletters Stratigr..

[52] Sliter WV (1989). Biostratigraphic zonation for Cretaceous planktonic foraminifers examined in thin section. J. Foraminifer. Res..

[53] Cobianchi M, Luciani V, Bosellini A (1997). Early Cretaceous nannofossils and planktonic foraminifera from northern Gargano (Apulia, southern Italy). Cretac. Res..

[54] Bellier, J. P., Moullade, M. & Huber, B. T. Mid-Cretaceous planktonic foraminifers from Blake Nose: revised biostratigraphic framework. *Proc. Ocean Drill. Program, Sci. Results* **171B**, 1–12 (2000).

[55] Coccioni R, Luciani V, Marsili A (2006). Cretaceous oceanic anoxic events and radially elongated chambered planktonic foraminifera: Paleoecological and paleoceanographic implications. Palaeogeogr. Palaeoclimatol. Palaeoecol..

[56] Koutsoukos EAM (1992). Late Aptian to Maastrichtian foraminiferal biogeography and palaeoceanography of the Sergipe Basin Brazil. Palaeogeogr. Palaeoclimatol. Palaeoecol..

[57] Kochhann KGD, Koutsoukos EAM, Fauth G, Sial AN (2013). Aptian-Albian planktic foraminifera from DSDP Site 364 (offshore Angola): Biostratigraphy, paleoecology, and paleoceanographic significance. J. Foraminifer. Res..

[58] Silva-Telles Jr., A. C. & Viana, M. S. S. Paleoecologia dos ostracodes da Formação Santana (bacia do Araripe): um estudo ontogenético de populações. in *1° Simpósio sobre a bacia do Araripe e bacias interiores do Nordeste* 309–328 (URCA, 1990).

[59] Do Carmo, D. A., Coimbra, J. C., Whatley, R. C., Antonietto, L. S. & De Paiva Citon, R. T. Taxonomy of limnic Ostracoda (Crustacea) from the Alagamar Formation, middle–upper Aptian, Potiguar Basin, northeastern Brazil. *J. Paleontol.* **87**, 91–104 (2013).

[60] Antonietto, L. S., Do Carmo, D. A., Viviers, M. C. & Adôrno, R. R. Biostratigraphic and paleozoogeographic review of the upper Aptian-Albian ostracods of Riachuelo Formation, Sergipe-Alagoas Basin, Northeastern Brazil. *Rev. Bras. Paleontol.* **18**, 355–368 (2015).

[61] Ramos MIF, Rossetti DF, Paz JDS (2006). Caracterização e significado paleoambiental da fauna de ostracodes da Formação Codó (Neoaptiano), leste da Bacia de Grajaú, MA Brasil. Rev. Bras. Paleontol..

[62] biostratigraphic and paleogeographic implications (2008). Do Carmo, D. A., Whatley, R., de Queiroz Neto, J. V. & Coimbra, J. C. On the validity of two Lower Cretaceous non-marine ostracode genera. J. Paleontol..

[63] Piovesan EK, Nicolaidis DD, Fauth G, Viviers MC (2013). Ostracodes from the Aptian e Santonian of the Santos, Campos and Espírito Santo basins Brazil. J. South Am. Earth Sci..

[64] Tomé, M. E. & Lima Filho, M. F. Ostracodes da Bacia de Cedro, Andar Alagoas, Estado de Pernambuco, NE, Brasil: implicações paleoambientais e bioestratigráficas. *Estud. Geológicos* **20**, 49–63 (2010).

[65] Souza D, Piovesan E, Neumann VHML (2017). Ostracodes do Aptiano-Albiano da Bacia do Araripe: Implicações paleoambientais e bioestratigráficas. Estud. Geológicos.

[66] Regali, M. da S. P. & Viana, C. F. *Sedimentos do Neojurássico-Eocretáceo do Brasil: idade e correlação com a escala internacional*. (Petrobras, 1989).

[67] Regali, M. da S. P. & Silva Santos, P. R. da. Palinoestratigrafia e geocronologia dos sedimentos albo–aptianos das Bacias de Sergipe e de Alagoas - Brasil. *Bol. do 5° Simpósio sobre o Cretáceo do Bras.* 411–419 (1999).

[68] Rios-Netto, A. D. M., Regali, M. D. S. P., Carvalho, I. D. S. & Freitas, F. I. De. Palinoestratigrafia do intervalo Alagoas da Bacia do Araripe, Nordeste do Brasil. *Rev. Bras. Geociencias* **42**, 331–342 (2012).

[69] Teixeira MC, Mendonça Filho JG, de Oliveira AD, Assine ML (2017). Faciologia orgânica da Formação Romualdo (Grupo Santana, Cretáceo Inferior da Bacia do Araripe): caracterização da matéria orgânica sedimentar e interpretação paleoambiental. Geol. USP. Série Científica.

[70] Varejão FG (2016). Upper Aptian mixed carbonate-siliciclastic sequences from Tucano Basin, Northeastern Brazil: Implications for paleogeographic reconstructions following Gondwana break-up. Cretac. Res..

[71] Marques FO, Nogueira FCC, Bezerra FHR, de Castro DL (2014). The Araripe Basin in NE Brazil: an intracontinental graben inverted to a high-standing horst. Tectonophysics.

[72] Premoli Silva, I. & Boersma, A. Cretaceous planktonic foraminifers-DSDP Leg 39 (South Atlantic). *Initial Reports Deep Sea Drill. Proj.* **39**, 615–641 (1977).

[73] Caron M (1978). Cretaceous planktonik foraminifers from DSDP Leg 40, southeastern Atlantic Ocean. Initial Rep. Deep Sea Drill. Proj..

[74] Viviers MC (1987). Foraminíferos planctônicos no Cretáceo médio da Bacia de Santos Brasil. Rev. Bras. Geociências.

[75] Longoria, J. F. Stratigraphic, morphologic and taxonomic studies of Aptian planktonic foraminifera. *Rev. Esp. Micropaleontol.* **Special is**, 1–162 (1974).

[76] Leckie, R. M. Mid-Cretaceous planktonic foraminiferal biostratigraphy off central Morocco, Deep Sea Drilling Project Leg 79, Sites 545 and 547. *Initial reports DSDP, Leg 79, Las Palmas to Brest* 579–620 (1984) 10.2973/dsdp.proc.79.122.1984.

[77] Koutsoukos EAM, Mello MR, De Azambuja Filho NC, Hart MB, Maxwell JR (1991). The upper Aptian-Albian succession of the Sergipe Basin, Brazil: an integrated paleoenvironmental assessment. Am. Assoc. Pet. Geol. Bull..

[78] Koutsoukos EA, Bengtson P (1993). Towards an integrated biostratigraphy of the upper Aptian-Maastrichtian of the Sergipe Basin Brazil. Doc. des Lab. Géologie.

[79] Moullade, M., Tronchetti, G. & Bellier, J.-P. The Gargasian (Middle Aptian) strata from Cassis-La Bédoule (lower Aptian historical stratotype, SE France): planktonic and benthic foraminiferal assemblages and biostratigraphy. *Carnets géologie (Notebooks Geol.* (2005) 10.4267/2042/1460.

[80] Banner F, Copestake P, White MR (1993). Barremian-Aptian Praehedbergellidae of the North Sea area: a reconnaisance. Bull. Nat. Hist. Museum Lond..

[81] BouDagher-Fadel, M. K., Banner, F. T., Gorbachik, T. N., Simmons, M. D. & Whittaker, J. E. Evolution in the Early Cretaceous planktonic foraminiferal genus Blefuscuiana. *Neues Jahrb. fur Geol. und Palaontologie - Abhandlungen* **201**, 243–258 (1996).

[82] Bolli HM (1959). Planktonic foraminifera from the Cretaceous of Trinidad B. W. I.. Bull. Am. Paleontol..

[83] BouDagher-Fadel, M. K., Banner, F. T. & Whittaker, J. . E. *The early evolutionary history of planktonic Foraminifera*. *British Micropalaeontological Society Publication Series* (British Micropalaeontological Society, 1997). 10.2307/1486073.

[84] Boudagher-Fadel, M. K. *Biostratigraphic and geological significance of planktonic Foraminifera*. *Developments in Palaeontology and Stratigraphy* (University College London, 2015). 10.1016/B978-0-444-53638-9.00001-5.

[85] Coccioni R, Erba E, Premoli-Silva I (1992). Barremian-Aptian calcareous plankton biostratigraphy from the Gorgo Cerbara section (Marche, central Italy) and implications for plankton evolution. Cretac. Res..

[86] Coccioni R (1998). Integrated stratigraphic, palaeontological, and geochemical analysis of the uppermost Hauterivian Faraoni Level in the Fiume Bosso section, Umbria-Marche Apennines Italy. Cretac. Res..

[87] Koutsoukos, E. A. M. Evaluating the evidence on the opening of the Equatorial Atlantic Gateway and its global impact. in *Geological Society of America Abstracts with Programs* vol. 39 445 (Geological Society of America, 2007).

[88] Dias-Brito D (2000). Global stratigraphy, palaeobiogeography and palaeoecology of Albian-Maastrichtian pithonellid calcispheres: Impact on Tethys configuration. Cretac. Res..

[89] Bengtson, P., Koutsoukos, E. A. M., Kakabadze, M. V & Zucon, M. H. Ammonite and foraminiferal biogeography and the opening of the Equatorial Atlantic Gateway. in *1er Symposium International de Paléobiogéographie* 12 (Université Pierre et Marie Crurie, 2007).

[90] Varejão F (2019). Microbialite fields developed in a protected rocky coastline: the shallow carbonate ramp of the Aptian Romualdo Formation (Araripe Basin, NE Brazil). Sediment. Geol..

[91] Benson, R. *et al.* Systematic descriptions. in *Treatise on Invertebrate Paleontology* (eds. Moore, R. C. & Pitrat, C. W.) . Q99–Q421 (Geological Society of America and University of Kansas Press, 1961).

[92] Anderson FW (1985). Ostracod faunas in the Purbeck and Wealden of England. J. Micropalaeontol.

[93] Rouchy JM, Camoin G, Casanova J, Deconinck JF (1993). The central palaeo-Andean basin of Bolivia (Potosi area) during the late Cretaceous and early Tertiary: reconstruction of ancient saline lakes using sedimentological, paleoecological and stable isotope records. Palaeogeogr. Palaeoclimatol. Palaeoecol..

[94] Horne DJ (2002). Ostracod biostratigraphy and palaeoecology of the Purbeck Limestone Group in Southern England. Spec. Pap. Palaeontol..

[95] Van Doninck K, Schön I, Maes F, De Bruyn L, Martens K (2003). Ecological strategies in the ancient asexual animal group Darwinulidae (Crustacea, Ostracoda). Freshw. Biol..

[96] Sousa AJ, Carvalho IS, Ferreira EP (2018). Western Gondwana non-marine ostracods from Early Cretaceous low-latitude ephemeral lake, Northeastern Brazil. J. South Am. Earth Sci..

[97] Scholle, P. A. & Ulmer-scholle, D. S. *A color guide to the petrography of carbonate rocks - grains, textures, porosity, diagenesis*. (American Association of Petroleum Geologists, 2003).

[98] Hesse R, Schacht U (2011). Early diagenesis of deep-sea sediments. Dev. Sedimentol..

[99] Flügel E (2004). Microfacies of carbonate rocks. Microfacies Carbon. Rocks.

[100] Huggett, J. M. Glauconites. in *Reference Module in Earth Systems and Environmental Sciences* 542–548 (2019). 10.1016/b978-0-12-409548-9.11978-5.

[101] Jones, R. & Charnock, M. Morphogroups of agglutinating foraminifera. Their life positions and feeding habits and potential applicability in (paleo)ecological studies. *Revue de Paléobiologie* vol. 4 311–320 (1985).

[102] Kaminski, M. A. & Kuhnt, W. Tubular agglutinated foraminifera as indicators of organic carbon flux. in *Proceedings of the Fourth International Workshop on Agglutinated Foraminifera 1993* (eds. Kaminski, M., Geroch, S. & Gasinski, M.) 141–144 (Grzybowski Foundation Special Publication no. 3, 1995).

[103] Kaminski, M. A., Neagu, T., Platon, E. & Wcie, L. A revision of the Lower Cretaceous foraminiferal genus *Falsogaudyinella* from northwest Europe and Romania, and its relationship to *Uvigerinammina*. in *Proceedings of the Fourth International Workshop on Agglutinated Foraminifera 1993* (eds. Kaminski, M., Geroch, S. & Gasinski, M.) 145–157 (Grzybowski Foundation Special Publication no. 3, 1995).

[104] Gradstein FM, Kaminski MA, Agterberg FP (1999). Biostratigraphy and paleoceanography of the Cretaceous seaway between Norway and Greenland. Earth Sci. Rev..

[105] Pessoa Neto ODC (2007). Bacia Potiguar. Bol. Geociências da Petrobras.

[106] Do Carmo, D. A., Sanguinetti, Y. T., Coimbra, J. C. & Guimarães, E. M. Paleoecologia dos ostracodes nao-marinhos do Cretaceo Inferior da Bacia Potiguar, RN, Brasil. in *5° Simpósio sobre o Cretáceo do Brasil* 383–391 (UNESP, 1999).

[107] Do Carmo, D. A. *et al.* Palaeoenvironmental assessment of Early Cretaceous limnic ostracods from the Alagamar Formation, Potiguar Basin, NE Brazil. *Cretac. Res.* **85**, 266–279 (2018).

[108] Tappan H (1940). Foraminifera from the Grayson Formation of northern Texas. J. Paleontol..

[109] Coccioni R (2014). The neglected history of oceanic anoxic event 1b: Insights, new data from the Poggio le Guaine section (Umbria-Marche Basin). Stratigraphy.

[110] Ferraro S, Coccioni R, Sabatino N, Del Core M, Sprovieri M (2020). Morphometric response of late Aptian planktonic foraminiferal communities to environmental changes: a case study of Paraticinella rohri at Poggio le Guaine (central Italy). Palaeogeogr. Palaeoclimatol. Palaeoecol..

[111] Sabatino N (2018). Mercury anomalies in upper Aptian–lower Albian sediments from the Tethys realm. Palaeogeogr. Palaeoclimatol. Palaeoecol..

[112] Premoli Silva, I., Soldan, D. M. & Petrizzo, M. R. Upper Hauterivian–upper Barremian planktonic foraminiferal assemblages from the Arroyo Gilico section (Southern Spain). *J. Foraminifer. Res.* **48**, 314–355 (2018).

[113] Leckie RM (1987). Paleoecology of mid-Cretaceous planktonic foraminifera: a comparison of open ocean and epicontinental sea assemblages. Micropaleontology.

[114] Leckie, R. M. Middle Cretaceous planktonik foraminifers of the Antarctic margin: Hole 693A, ODP Leg 113. in *Proceedings of the Ocean Drilling Program, Scientific Results* (eds. Barker, P. F., Kennett, J. P. & Others) vol. 113 319–324 (College Station, 1990).

[115] Carvalho MDA, Bengtson P, Lana CC (2016). Late Aptian (Cretaceous) paleoceanography of the South Atlantic Ocean inferred from dinocyst communities of the Sergipe Basin Brazil. Paleoceanography.

[116] Arai, M., Coimbra, J. C. & Silva-Telles Jr., A. C. Síntese Bioestratigráfica da Bacia do Araripe (Nordeste do Brasil). in 2° Simpósio sobre a Bacia do Araripe e bacias interiores do Nordeste (eds. Barros, L. M., Nuvens, P. C. & Filgueira, J. B.) vol. 2 109–124 (2001).

[117] Schaller H (1969). Revisão estratigráfica da Bacia de Sergipe/Alagoas. Bol. Técnico da Petrobras.

[118] Viviers MC, Koutsoukos EAM, Silva-Telles AC, Bengtson P (2000). Stratigraphy and biogeographic affinities of the late Aptian-Campanian ostracods of the Potiguar and Sergipe basins in northeastern Brazil. Cretac. Res..

[119] Poropat SF, Colin JP (2012). Early cretaceous ostracod biostratigraphy of eastern Brazil and western Africa: an overview. Gondwana Res..

[120] Ogg, J. G., Hinnov, L. A. & Huang, C. Cretaceous. in The Geologic Time Scale 2012 (eds. Gradstein, F. M., Ogg, J. G., Schmitz, M. D. & Ogg, G. M.) vol. 2 793–853 (Elsevier, 2012).

