# Supplementary material for: New marine data and age accuracy of the Romualdo Formation, Araripe Basin, Brazil
Source: Sci Rep. 2020 Sep 25;10:15779. doi: 10.1038/s41598-020-72789-8 (PMC7519656; doi:10.1038/s41598-020-72789-8)
Supplement: Supplementary file 1 [file 41598_2020_72789_MOESM1_ESM.docx]

**NEW MARINE DATA AND AGE ACCURACY OF THE ROMUALDO FORMATION, ARARIPE BASIN, BRAZIL**

Robbyson Mendes Melo1*, Juliana Guzmán1+, Débora Almeida1, Enelise Katia Piovesan1+, Virgínio Henrique de Miranda Lopes Neumann1+ and Ariany de Jesus e Sousa2+

1 Laboratory of Applied Micropaleontology (LAGESE / LITPEG), Department of Geology, Univesity Federal of Pernambuco, Recife-PE, Brazil

2 PETROBRAS/CENPES/PDGEO/BPA, Ilha do Fundão, Rio de Janeiro-RJ, Brazil

*[corresponding:](mailto:corresponding.author@email.example) robbysonmelo@gmail.com

+these authors contributed equally to this work

# ABSTRACT

A combined biostratigraphic and palaeoecological study of foraminifera, ostracodes and microfacies was carried out on the Aptian in the Sítio Sobradinho section of the Araripe Basin, northeast Brazil. The analysed section represents a deepening-upward sequence with mid-ramp shoal and outer ramp to basin facies associations on a mixed siliciclastic-carbonate marine ramp. The analysed rocks are dominated by Early Cretaceous planktic foraminifera (*Hedbergella aptiana, H. praelippa, H. sigali*, *Blesfucuiana* cf. *cumulus, Microhedbergella miniglobularis, Gorbachikella* cf. *kugleri, Pseudoguembelitria blakenosensis, Globigerinelloides clavatus*, *Globigerinelloides* aff. *aptiensis*, *Gubkinella* sp. and *Loeblichella* sp.). Ostracoda fauna is composed mainly of *Pattersoncypris crepata* and *Pattersoncypris micropapillosa*. The occurrence of *P*. *crepata* associated with the Aptian planktic foraminifera demonstrates the potential of this ostracode species to date this interval. The planktic foraminifera from the upper Aptian (*Microhedbergella miniglobularis* Zone) of the Araripe Basin show characteristical Tethyan affinities.

**Appendix A**. Supplementary data: taxonomic reference list.

Taxa listed in alphabetical order.

**Planktic** **foraminifera=** the taxonomic concepts used in this study follow Huber and Leckie (2011) and Petrizzo et al. (2014), Coccione et al. (2014) and the Mikrotax online Mesozoic Planktic Foraminifera Dictionary (<http://www.mikrotax.org/>.).

*Globigerinelloides* aff. *aptiensis* = *Globigerinelloides aptiensis* Longoria, 1974

*Globigerinelloides clavatus* Verga and Premoli Silva, 2005

*Gorbachikella* cf. *kugleri* (Bolli, 1959) = *Globigerina kugleri* Bolli, 1959

*Gubkinella* sp. = *Gubkinella* Suleymanov, 1955 = specimens which cannot be assigned to established species

*Hedbergella aptiana* Bartenstein, 1965

*Blefuscuiana cumulus* (Banner, Copestake and White, 1993) = *Blefuscuiana excelsa cumulus* Banner, Copestake and White, 1993

*Hedbergella* *praelippa* Huber and Leckie, 2011

*Hedbergella* *sigali* Moullade, 1966

*Loeblichella* sp. = *Loeblichella* Pessagno, 1967 = specimens which cannot be assigned to established species

*Microhedbergella miniglobularis* Huber and Leckie, 2011

*Microhedbergella renilaevis* Huber and Leckie, 2011

*Microhedbergella rischi* (Moullade, 1974) emend. Huber and Leckie, 2011 *= Hedbergella rischi* Moullade, 1974

*Paraticinella rohri* (Bolli, 1959) emend. Ando, Huber and Premoli Silva 20013 = *Ticinella roberti eubejaouaensis* Randrianasolo and Anglada 1998

*Pseudoguembelitria blakenosensis* Huber and Leckie, 2011

**Benthic foraminifera =** the taxonomic concepts used in this study follow Loeblich and Tappan (1988), Kaminski et al. (1995) and Bolli et al (1994).

*Ammobaculites* Cushman, 1910

*Astacolus* Montfort, 1808

*Bathysiphon* Sars, 1872

*Falsogaudriynella* cf. *tealbyensis* = *Gaudryinella tealbyensis* Bartenstein, 1956

*Globulina* d'Orbigny, 1839

*Glomospira charoides* Jones and Parker, 1860

*Lenticulina* Lamarck, 1804

*Lingulogavelinella* Malapris, 1965

*Pyramidulina* Fornasini, 1894

*Rhizammina* Brady, 1879

**Ostracoda =** the taxonomic concepts used in this study follow Ellis & Messina (1940 et seq) and Do Carmo et al. (2013).

*Alicenula* Rossetti and Martens, 1998

*Aracajuia* Krömmelbein, 1967

*Cytherella* Jones, 1849

*Cytherelloidea* Alexander, 1929

*Patellacythere* Gründel and Kozur, 1972

*Damonella* Anderson, 1966

*Ilyocypris* Brady and Norman, 1889

*Pattersoncypris crepata* (Do Carmo et al., 2013) = *Harbinia crepata* Do Carmo et al., 2013

*Pattersoncypris micropapilosa* Bate, 1972

*Pattersoncypris* Bate, 1972

**References**

Coccioni, R. *et al.* The neglected history of oceanic anoxic event 1b: Insights, new data from the Poggio le Guaine section (Umbria-Marche Basin). *Stratigraphy* **11**, 245–282 (2014).

Do Carmo, D. A. *et al.* Palaeoenvironmental assessment of Early Cretaceous limnic ostracods from the Alagamar Formation, Potiguar Basin, NE Brazil. *Cretac. Res.* **85**, 266–279 (2018).

Ellis, B. F. & Messina, A. R. Catalogue of Ostracoda. *Micropaleontology Press*, American Museum of Natural History, New York (1940 et seq).

Huber, B. T. & Leckie, R. M. Planktic Foraminiferal Species Turnover Across Deep-Sea Aptian/ Albian Boundary Sections. *J. Foraminifer. Res.* **41**, 53–95 (2011).

Kaminski, M. A., Neagu, T. & Platon, E. A revision of *Falsogaudryinella* from the Lower Cretaceous of the North Sea and Romania, and its relationship to *Uvigerinammina*. in: *Proceedings of the Fourth International Workshop on Agglutinated Foraminifera* (eds. Kaminski, M. A. et al.) **3,** 145–157 (Grzybowski Foundation Special Publication, 1995).

Loeblich Jr., A. R. & Tappan, H. Foraminiferal Genera and Their Classification. *van Nostrand Reinhold Company*, New York, 970 p. (1988).

Petrizzo, M. R., Huber, B. T., Gale, A. S., Barchetta, A. & Jenkyns, H. C. Abrupt planktic foraminiferal turnover across the niveau kilian at col de pré-guittard (Vocontian Basin, southeast France): New criteria for defining the Aptian/Albian boundary. *Newsletters Stratigr.* **45**, 55–74 (2012).
